# COVID-19 Outbreak: An Overview on Dentistry

**DOI:** 10.3390/ijerph17062094

**Published:** 2020-03-22

**Authors:** Gianrico Spagnuolo, Danila De Vito, Sandro Rengo, Marco Tatullo

**Affiliations:** 1 Department of Neurosciences, Reproductive and Odontostomatological Sciences, University of Naples “Federico II”, Via Pansini 5, 80131 Naples, Italy; 2 Department of Basic Medical Sciences, Neurosciences and Sense Organs, University of Bari “Aldo Moro”, 70124 Bari, Italy; danila.devito@uniba.it (D.D.V.); sanrengo@unina.it (S.R.); marco.tatullo@tecnologicasrl.com (M.T.); 3 Marrelli Health—Tecnologica Research Institute, Biomedical Section, Street E. Fermi, 88900 Crotone, Italy

**Keywords:** COVID-19, dentistry, SARS-CoV-2, infection, coronavirus

Coronavirus disease 2019, also called COVID-19, is the latest infectious disease to rapidly develop worldwide. COVID-19 has as its etiologic agent the severe acute respiratory syndrome coronavirus 2 (SARS-CoV-2): the 2019 coronavirus is different from SARS-CoV, but it has the same host receptor: human angiotensin-converting enzyme 2 (ACE2). SARS-CoV-2 was first discovered in 2019 in Wuhan, China, unfortunately spreading globally, resulting in the 2019–2020 pandemic, as declared by the World Health Organization (WHO) and the Public Health Emergency of International Concern (PHEIC). The infection started in Asia, but it has rapidly spread across the world: according to the WHO, this is the first pandemic caused by a coronavirus. Against this landscape, the treatment of COVID-19 is based on containment measures: in China and South Korea, the severe application of such interventions has regularly and drastically reduced new cases, and this experience shows that a reversion of epidemic growth is possible in the short-term.

On the other hand, in Italy, the reported cases have grown impressively over time, leading to the country obtaining a prominent position in the international scenario of the infected patients. This emerging pandemic and its severe outbreak in the Italian population have induced the Italian Government first and then the European Union to promote drastic impact measures to “flatten the curve” of the COVID-19 infection and in turn avoid health systems (in particular, intensive care units) being overwhelmed, resulting in fewer deaths [1]. The limitation of people circulating outside their home, social distancing, the cessation of almost all working activities and the request to the population to use protective masks and gloves all have the aim of minimizing the likelihood that people who are not infected come into contact with others who are already infected and probably still asymptomatic [2]. As always happens, healthcare professionals have been immediately involved in the national emergency, working hard, often day and night: unfortunately, small numbers of them have also become infected, and some have tragically died. Dentists are often the first line of diagnosis, as they work in close contact with patients. On 15 March 2020, the New York Times published an article entitled “The Workers Who Face the Greatest Coronavirus Risk”, where an impressive schematic figure described that dentists are the workers most exposed to the risk of being affected by COVID-19, much more than nurses and general physicians [3]. To take significant actions against this harmful disease, the American Dental Association updated its webpage in March, including a link to frequently asked questions from member dentists covering topics such as personal protective equipment and patient communications. Recently, an interesting paper written by researchers from Wuhan University School and Hospital of Stomatology was published with several recommendations for dentists and dental students to manage COVID-19 patients [4]. Dentists have been recommended to take several personal protection measures and avoid or minimize operations that can produce droplets or aerosols; moreover, the use of saliva ejectors with a low volume or high volume can reduce the production of droplets and aerosols. Taking into consideration the severity of the pandemic COVID-19, and in the light of the massive commitment of several dental associations and the most prestigious dental journals, it is essential to give clear and easy guidelines to manage dental patients and to make working dentists safe from any risk. A fundamental concept is that the transmission of the virus is mainly through inhalation/ingestion/direct mucous contact with saliva droplets; it is also critical to remember that the virus can survive on hands, objects or surfaces that were exposed to infected saliva in the previous nine days [4,5]. Since the viral load contained in the human saliva is very high, rinses with antiseptic mouthwashes can only reduce the infectious amount but are not able to eliminate the virus in the saliva [4,5]. In this light, a few important concepts would be useful to briefly report and discuss here. 

The most recommended guidelines indicate that dentists should avoid the scheduling of any patient: only such urgent dental diseases can be considered during the COVID-19 outbreak. This action will drastically limit the interpersonal contact, the waiting time of patients in dental cabinets and, in general, the conditions predisposing patients to be infected. When the dentists treat patients, they should intercept the potentially infected person before they reach the operating areas; for example, those with a fever measuring >37.5 °C and the posing of a few questions about the patient’s general health status in the last 7 days, and about the risk of having been in contact with other infected persons. 

The management practice of the operating area should be quite similar to what happens with other patients affected by infectious and highly contagious diseases. As often as possible, the staff should work at an adequate distance from patients; furthermore, handpieces must be equipped with anti-reflux devices to avoid contaminations, improving the risk of cross-infections. Finally, during the operating sessions, the dentist should prefer procedures reducing the quantity of aerosol produced in the environment [4,5]. 

Personal prevention, both for health personnel and for patients, must be associated with the prevention of the spread of the virus through environmental remediation. In particular, due to the high proliferation of the virus in the particles exhaled by coughing and sneezing, every surface in the waiting room must be considered at risk; therefore, in addition to providing adequate periodic air exchange, all surfaces, chairs, magazines, and doors that come into contact with healthcare professionals and patients must be considered “potentially infected”. It may be useful to make an alcoholic disinfectant and masks available to patients in the waiting room. The entire air conditioning system must be sanitized very frequently [4,5].

A recent study indicates that copper and paper can allow the virus to survive for 4 to over 24 hours. On the other hand, the infectious charge can be drastically reduced only after at least 48 hours for steel and 72 hours for plastic [6]. Therefore, the virus remains longer on steel instruments, or disposable material exposed to the flows of contaminated air, than in a magazine in the waiting room. In light of this reflection, the substantial action to be taken is to promote maximum hand and surface hygiene, given that the virus is completely inactivated by water, soap, and other detergents.

In conclusion, the significant limitation of clinical and surgical activities in the medical and dental sector has represented a very impactful measure on the economy of the sector. Nevertheless, this drastic intervention has made it possible to protect the health and safety of citizens and contain the expansion of the coronavirus. Therefore, the policies and measure packages adopted by governments are addressed to all dental associations, stating clear guidelines to prevent and to control COVID-19 infection in oral diagnosis and treatment in daily practice until a vaccine or a drug becomes available.
